# Enhanced Bioactivity of Pomegranate Peel Extract following Controlled Release from CaCO_3_ Nanocrystals

**DOI:** 10.1155/2022/6341298

**Published:** 2022-02-12

**Authors:** Francesca Baldassarre, Viviana Vergaro, Federica De Castro, Francesca Biondo, Gian Paolo Suranna, Paride Papadia, Francesco P. Fanizzi, Domenico Rongai, Giuseppe Ciccarella

**Affiliations:** ^1^Department of Biological and Environmental Sciences, UdR INSTM of Lecce University of Salento, Via Monteroni, 73100 Lecce, Italy; ^2^Institute of Nanotechnology, CNR NANOTEC, Consiglio Nazionale Delle Ricerche, Via Monteroni, 73100 Lecce, Italy; ^3^Department of Civil,Environmental,Land,Building Engineering and Chemistry (DICATECh), Politecnico di Bari, Via Orabona 4, Bari 70125, Italy; ^4^CREA-IT PE-Research Centre for Engineering and Agro-Food Processing, Via Lombardia, Pescara, Italy

## Abstract

Pomegranate peel extract is rich of interesting bioactive chemicals, principally phenolic compounds, which have shown antimicrobial, anticancer, and antioxidative properties. The aim of this work was to improve extract' bioactivity through the adsorption on calcium carbonate nanocrystals. Nanocrystals revealed as efficient tools for extract adsorption reaching 50% of loading efficiency. Controlled release of the contained metabolites under acidic pH has been found, as it was confirmed by quantitative assay and qualitative study through NMR analysis. Specific functionality of inorganic nanocarriers could be also tuned by biopolymeric coating. The resulting coated nanoformulations showed a great antimicrobial activity against *B. cinerea* fungus preventing strawberries disease better than a commercial fungicide. Furthermore, nanoformulations demonstrated a good antiproliferative activity in neuroblastoma and breast cancer cells carrying out a higher cytotoxic effect respect to free extract, confirming a crucial role of nanocarriers. Finally, pomegranate peel extract showed a very high radical scavenging ability, equal to ascorbic acid. Antioxidant activity, measured also in intracellular environment, highlighted a protective action of extract-adsorbed nanocrystals twice than free extract, providing a possible application for new nutraceutical formulations.

## 1. Introduction

*Punica granatum* is one of the fruits that most attracted the interest of researchers for its high potential use in medicine and food industry [1–3]. It is rich in several metabolites with antimicrobial, anticancer, antiobesity, antidiabetic, antiulcerogenic, and antihypertensive proprieties [4]. The above beneficial properties are not only limited to the edible part of fruit but also nonedible fractions (i.e., peel, seeds, flowers, bark, buds, and leaves) contain these compounds, and they come in huge quantities as by-products in pomegranate juice processing industries, constituting a great bioresource [5–7]. In particular, the peels represent about 50% of total fruit weight and are most often discarded as waste without any valorisation [8]. Interestingly, pomegranate peel extract contains the highest concentration of phytochemicals, principally phenolic compounds family, such as tannins, ellagitannins, and anthocyanins, including ellagic acid and punicalagin [9, 10]. These bioactive ingredients have proven antimicrobial propriety toward many pathogens and antiproliferative activity for different cell lines [11–13]. Recently, phenols from hydromethanol pomegranate (*Punica granatum* L.) peel extract have showed interesting antihyperglycemic, antihyperlipidemic, and antioxidant properties [14]. The smart recovery of pomegranates rind for production of extract could have multiple applications in different fields, such as biomedical, nutraceutical, and biocontrol [6, 13, 15]. There is a growing interest in finding natural antimicrobial compounds as a valid and safe alternative to conventional chemical treatments for managing plants infections and postharvest fruit diseases [3]. More than 19000 different types of fungi could affect crops causing serious economic losses in hundreds of countries around the world, such as *Phytophthora* [16], *Fusarium oxysporum* for potatoes and tomatoes [17], *Magnaporthe oryzae* for rice [18], and *Botrytis cinerea* for different fruits [19]. Furthermore, postharvest phase is very crucial for fresh fruits and vegetables, resulting in substantial economic losses up to 25%, due to various pathogens. The effective control still derives from agrochemicals use that negatively affects food, animals, environment, and human health. The presence of residues on food and drinking water cause toxicological side effects such as carcinogenicity and teratogenicity. The current challenge is to reduce the high persistence of pesticides as well as to fight pests' emergence paving the way for alternative strategies such as new biopesticides [20]. Since penicillin discovery, utilization of natural compounds to control pathogens has become crucial not only in medicine but also as ecofriendly strategy for crops protection. There are included plant-incorporated protectants, botanical and microbial-derived chemicals, and also synthetic analogues [18, 21, 22]. Recent research has been focused on essential oils or complex mixtures plants extracts, composed of several secondary metabolites which act in an interesting synergistic way preventing resistance phenomena [23]. Several plants extracts have been obtained, characterized, and investigated as biopesticides [24, 25]. *Punica granatum* peel extract showed high efficiency as natural pesticide to control phytopathogens of crops and postharvest fruits diseases preventing and treating bacteria, fungi, and parasites [26–30]. The high level of phenolic compounds provides the extract' antimicrobial effect reaching the same antifungal activity of isolated compound punicalagin that is characteristic of pomegranate [31, 32]. This bioactivity has been exploited to obtain new food preservatives and innovative materials for active packaging [15, 33–35]. Furthermore, the great antimicrobial propriety of pomegranate peel extract regards also different pathogens affecting human health, including foodborne pathogens, both Gram-positive and Gram-negative bacteria, *Streptococcus mutans* causes dental caries and fungi, as *Candida* species [12, 13, 36, 37]. The antiviral effect against many viruses was described, demonstrating the mechanism of interaction between polyphenols and viral capsid proteins [38, 39]. Since ancient times, potential biomedical applications of pomegranate are multiple; in fact, it is one of the most used plants in folk medicine. Its bioactivities include anti-inflammatory and antiproliferative effects which are derived on the modulation of different molecular targets. Literature is rich of studies about anticancer propriety of pomegranate against several cancer cell lines, also in clinicopathological studies [1, 9]. *Punica granatum* peel extract' nutraceutical benefits comprise preventive action toward a wide range of diseases and prebiotic potentiality to treat obesity [11, 40–42]. Therefore, the smart recovery of pomegranate peel to produce bioactive extract is very advantageous since it covers a wide range of applications. However, phytochemicals have some drawbacks, such as volatility, poor water solubility, and chemical-physical instability, which could limit their bioactivity. Nanoencapsulation and nanomaterials integration are suitable strategies to overcome these problems [43–46]. These approaches have been widely exploited for cancer therapeutics in nanomedicine that laid the foundations for new nutraceuticals and agrinanotechnology [47–51]. Recently, polymeric and solid lipid nanoparticles have been investigated for loading and controlled release of pomegranate extract or individual polyphenols (such as punicalagin and ellagic acid) improving the antiproliferative effect, obtaining nanochemoprevention of different cancers [52–55]. Furthermore, the combination with metal salts and phospholipids has been exploited to enhance, respectively, the antiviral effect and bioavailability [39, 56]. Biogenic synthesis has been exploited to improve antimicrobial activity of metallic nanoparticles through phytochemicals contributed. Novel bioinsecticides have been obtained through nanoencapsulation of cytotoxic plant extracts. By that time, nanomaterials application in agriculture and food is clearly in the research and development and marketable stage, thanks to nanomedicine reached results [57].

The present work relates to the adsorption of aqueous pomegranate peel extract on inorganic nanocarriers tuning their functionalities through biopolymeric coating. In particular, CaCO_3_ nanocrystals (nanoCaCO_3_) have been chosen, investigating chitosan coating to modulate compounds release and bioactivity. NanoCaCO_3_ previously showed their potential as delivery systems with a great affinity toward different biomolecules and drugs, interacting both with human cells and bacteria [58–60]. Aqueous pomegranate peel extract, from *P. granatum var. dente di cavallo* (PAE) [17], has been efficiently loaded on nanoCaCO_3_, and adsorption/release valuations have been supported by NMR analysis. Antiproliferative activity has been studied toward two human model cancer cell lines, and antimicrobial activity has been investigated for two aggressive plants/fruits pathogenic fungi. Antioxidant activity has been studied in in vitro assays. The versatile nature of CaCO_3_ nanocrystals and chitosan has been confirmed. Our results demonstrated the great potential of PAE nanoformulation based on bioinorganic nanosystems in different biological applications, ranging from human health and crops protection.

## 2. Materials and Methods

### 2.1. Plants Materials and Pomegranate Peel Extraction

Strawberry fruits (*Fragaria x ananassa* Duch.) used in the test came from a commercial organic market. All fruits were of the same size, without defects and with the same degree of ripeness. Samples of *P. granatum var. dente di cavallo*, given by the botanical garden at “CRA-FRUT,” Rome, Italy, were used. The powder of extract was obtained according to the method previously described [17]. Pomegranate peel was cut into small pieces and added to solvent: 80% of water (bidistilled water from a Milli-Q-System, Millipore, Bedford, UK) mixed with 20% of ethanol (analytical grade RPE, Carlo Erba Reagents, Milan, Italy). The mixture was sonicated for 15 min and then agitated overnight at 40°C. The ethanol was evaporated, and the extract obtained was centrifuged, and the supernatant filtered through the 0.45 *μ*m PTFE filter. The obtained pomegranate peel hydroalcoholic extract (in the following, PAE) was stored in a freezer at −20°C until further use.

### 2.2. Preparation of PAE-Adsorbed CaCO_3_ Nanocrystals

CaCO_3_ nanocrystals were synthesized as previous described [58, 61]. The adsorption of PAE on nanoCaCO_3_ was performed adding, drop by drop, a concentrated solution of PAE to an aqueous solution (pH 7.5) of nanoCaCO_3_ (100 mg) reaching the selected final concentrations (range 0.25–4.5%w/v). The nanoCaCO_3_ suspension was sonicated for 10 min prior loading. The physical adsorption was carried out mixing overnight the suspension at RT. After two washing, the resultant nanoCaCO_3_@PAE was dried in a stove at 50°C. Loading efficiency (LE) and loading capacity (LC) were calculated, keeping fixed the amount of particles and according to the following equations:

(1) $$\begin{array}{c} \%\text{LE}=100-\left(\frac{\left[\text{supernatant}\right]}{\left[\text{loading solution}\right]}\times 100\right), \end{array}$$

(2) $$\begin{array}{c} \%\text{LC}=\left(\frac{\text{mg adsorbed PAE}}{\text{mg }{\text{nanoCaCO}}_{3}}\right)\times 100. \end{array}$$

The quantification of PAE in supernatant has been obtained through spectrophotometric analysis, recording UV-vis absorption spectra at 260 nm by a Varian Cary 500 spectrophotometer. The unknown concentration was obtained referring to a standard curve using PAE solutions at known concentration in the range of 0.015–10%w/v and fitting the line through Origin software (Abs values have been multiplied by the dilution factor). The selected wavelength of 260 nm is the peak of PAE that corresponds to punicalagins adsorption peak. Washing solutions were also quantified to calculate the adsorbed mg and LC. PAE adsorption has been also valuated through a direct method performing thermogravimetric analysis (TGA) on nanoCaCO_3_@PAE powder. TGA has been carried out on aTA Instruments Q600 thermogravimetric analyser as previously reported [62, 63].

Chitosan-low MW (from Sigma Aldrich, Italy) has been used to investigate a polymeric coating on nanoCaCO_3_@PAE. The chitosan (CH) solution (1-2 mg/ml) was prepared by dissolving polymer in 0.1% glacial acetic acid and stirring for 24 h for complete dissolution. Coating has been obtained dispersing nanoCaCO_3_@PAE powder in CH solution and mixing overnight ad RT. After two washing, the resultant nanoCaCO_3_@PAE@CH were dried in a stove at 50°C.

### 2.3. Characterization of NanoCaCO_3_@PAE

Morphological analysis has been performed with transmission electronic microscopy (TEM) by a TEM microscope JEOL JEM1400Plus (Peabody, MA, USA), hydrodynamic diameter, and *ζ*-potential measurements were performed through the instrument Nano ZS90 (Malvern Instruments, Malvern, UK), as previous described [62].

Determination of antioxidant activity has been performed by neutralization of DPPH-free radicals (DPPH reagent from Sigma Aldrich, Italy). Briefly, 0.5 ml of samples (3 mg/ml of PAE and certain amounts of nanocrystals containing 3 mg/ml of PAE) was added to 2.5 ml of 0.1 mM DPPH-methanolic solution and vigorously shaken in the dark at room temperature. The absorbance of samples at 515 nm was measured after 30 min. Ascorbic acid and methanol were used as positive (standard) and negative controls, respectively. The antioxidant capacity (AC) of PAE, nanoCaCO_3_@PAE, and nanoCaCO_3_@PAE@CH solutions has been calculated using the following equation:

(3) $$\begin{array}{c} \text{AC}\left(\%\right)=\left[\frac{\left(\text{Abs control}-\text{Abs sample}\right)}{\text{Abs control}}\right]\times 100, \end{array}$$

where control is the DPPH-methanolic solution.

### 2.4. In Vitro PAE Release

*In vitro* PAE release profiles of nanoCaCO_3_@PAE and nanoCaCO_3_@PAE@CH were obtained by immersing a known quantity of samples into 1 ml of buffer solutions (pH 7.5 and pH 5) and were incubating at room temperature with constant agitation (900 rpm). After a predetermined period, nanoCaCO_3_ was precipitated through centrifugation, and released PAE was quantified according to the standard calibration curve as already described in previous paragraph. All release studies were performed in triplicate, and the means of all measurements were calculated. The results were presented in terms of cumulative percentage release as a function of time using the following formula:

(4) $$\begin{array}{c} \text{Cumulative percentage release}=\frac{W_{t}}{W_{l}}\times 100, \end{array}$$

where *W*_*t*_ is the amount of PAE released from nanocrystals at time *t* and *W*_*l*_ is the amount of adsorbed PAE as calculated by equation (2).

Release profiles have been also characterized by NMR analysis performing the same experiment in D_2_O.

### 2.5. NMR Measurements

All measurements were performed on a Bruker Avance III 600 Ascend NMR spectrometer (Bruker, Karlsruhe, Germany) operating at 600.13 MHz for ^1^H observation, equipped with cryoprobe, *z* axis gradient coil, and automatic tuning-matching (ATM). Experiments were run at 300 K. For each sample, a zgcppr Bruker standard pulse sequence was applied to suppress the residual water signal. A total of 64 scans (with 16 dummy scans) were collected into 64 k data points with a total spin-spin relaxation delay of 35 *µ*s. The FIDs were multiplied by an exponential weighting function corresponding to a line broadening of 0.3 Hz before Fourier transformation, phasing, and base line correction. All spectra were referenced to the trimethylsilyl propionate (TSP) signal (*δ* = 0.00 ppm) also used as internal standard for the quantitative comparisons. The metabolites were assigned through ^1^H 1D and 2D NMR spectra (^1^H-COSY, JRES, ^1^H-^13^C HSQC, and ^1^H-^13^C HMBC) analysis.

#### 2.5.1. Study of pH Dependence in the Release of PAE from NanoCaCO_3_

30 mg of nanoCaCO_3_@PAE were, respectively, weighted into two autoclaved 2 mL Eppendorf tube and resuspended into 600 *μ*L of D_2_O (pH 8.9). In order to evaluate the PAE release also at acidic condition, DCl was added to one of them to achieve a final pH of 5.01. For both samples (at acidic and basic pH), the ^1^H NMR spectra were collected and monitored for two weeks.

#### 2.5.2. Comparative Studies between NanoCaCO_3_@PAE and NanoCaCO_3_@PAE@CH

Equally PAE-loaded nanoCaCO_3_@PAE and nanoCaCO_3_@PAE@CH were incubated at different pH (acidic and basic) in D_2_O and left under stirring for 48 h. Then, nanoparticles were centrifuged, and supernatants were collected for the NMR analysis.

### 2.6. Fungal Strains and In Vitro Assays

*Botrytis cinerea* (strain CRA-DC Roma collection number 1623) and *Fusarium oxysporum f. sp. lycopersici* (strain CRA-PAV collection n. ER1372) with a good level of virulence were used. The fungi were kept on potato dextrose agar (PDA, Oxid CM 0139) and preserved at 4°C. As needed, the isolates were grown for 7-8 days on PDA in the dark at 24 ± 2°C.

Mycelia growth of *B. cinerea* and *F. oxysporum* were assessed by measuring radial growth on 90 mm Petri dishes, containing potato dextrose agar (PDA). In treated plates, 5, 10 and 20 mg of nanoCaCO_3_@PAE, nanoCaCO_3_@PAE@CH and PAE were added to 20 mL of PDA as the media was about to gel (50 ± 3°C) such that the concentration corresponded to 0.25, 0.5, or 1% (w/v), respectively. In untreated plates (negative control), samples contained PDA without extract. A plate containing a standard fungicide (Marisan 50 PB, Dichloran 60%, SIAPA, Milan, Italy) was used at the recommended concentration as a positive control. A 5 mm diameter plug of inoculum was taken from the actively growing margin of 7-day-old cultures of fungus and aseptically placed face up in the centre of each Petri plate. Radial growth was measured every day, starting three days after incubation at 24 ± 2°C and until the untreated plates were overgrown. Each treatment was replicated four times, and the test was repeated 30 days later.

### 2.7. Fruits Protection Assay

Strawberry fruits were submerged for 30 s in a 1 L of the respective solutions. Fruits dripping in water was used as the control. The fruits were then dried in air for 1 h and layer on the bottom of plastic boxes in groups of 15. After treatments, strawberries were stored first for 2 days at 2 ± 1°C, 95–98% RH, and then at 20 ± 1°C, 95–98% RH for 6 days (shelf life). Each treatment consisted of 60 fruits (15 fruits × 4 replicates) and included fruits treated with water as control; fruits treated with nanoCaCO_3_@PAE@CH at the concentration of 0.25%, 0.5%, and 1%; fruits treated with PAE at the concentration of 1%; and fruits treated with fungicide at the concentration of 0.5%. Disease severity (DS) was recorded on a 0–5 scale where 0 = no symptoms; 1 = 1–20% fruit surface infected; 2 = 21–40% fruit surface infected; 3 = 41–60% fruit surface infected; 4 = 61–80% fruit surface infected; and 5 = more than 81% of the strawberry surface infected and with sporulation. The percentage disease severity of each treatment was calculated by the formula: DS % = (sum of all disease ratings × 100)/(total number of ratings × maximum disease grade). Disease incidence (DI) was determined according to the formula: DI% = (no. of infected plants × 100)/(total no. of plants assessed). The test was repeated three times.

### 2.8. Human Cells Assays

#### 2.8.1. Cells Culture

Human breast cancer (MCF7) and human neuroblastoma (SH-SY5Y) cells were cultured in Dulbecco's modified eagle medium (DMEM; Sigma Aldrich, Darmstadt, Germany) supplemented with 10% fetal bovine serum (FBS; Sigma Aldrich, Darmstadt, Germany), 1% glutamine, and 1% penicillin/streptomycin (Invitrogen, Carlsbad, California, USA) in a humidified incubator at 37°C and 5% CO_2_ and 95% relative humidity.

#### 2.8.2. Cell Proliferation Assay

MCF7 and SH-SY5Y cells were treated for 24-48-72 h, with PAE at concentrations of 0.1 and 0.25 mg/ml and with nanoCaCO_3_@PAE and nanoCaCO_3_@PAE@CH at concentrations of 0.2 and 0.5 mg/ml in order to compare similar quantities of PAE. Cells were also treated with naked nanoCaCO_3_ as negative control. Cell proliferation was evaluated by MTT (3-(4,5-dimethylthiazol-2-yl)-2,5-diphenyltetrazolium bromide) assay [62].

### 2.9. Intracellular ROS/RNS Measurement Assays

The production of ROS, NO, and superoxide in SH-SY5Y and MCF7 cells was measured using the Cellular ROS/RNS Detection Assay Kit (Abcam, ab13947) following the manufacturer's protocol. Briefly, cells were treated for 24 h with PAE (0.1 mg/mg), nanoCaCO_3_@PAE (0.2 mg/ml), and nanoCaCO_3_@PAE@CH (0.2 mg/ml). Then, cells were washed with PBS and treated with 50 *μ*M of commercial H_2_O_2_ for 1 h to induce oxidative stress. Cells in medium only were used as positive control. Experiment has been performed also in absence of H_2_O_2_ treatment. Cells were treated for 30 minutes at 37°C in the dark with RNS detection solution. Cells were trypsinized and immediately subjected to cytofluorimeter measurements. The fluorescence was detected using three different lasers, at excitation/emission 490/520 nm (for ROS), another one at excitation/emission 520/600 nm (for superoxide), and one at excitation/emission 645/660 nm (for nitric oxide).

## 3. Results and Discussion

### 3.1. NanoCaCO_3_-Based Formulation of PAE: Adsorption and Release Study

**Figure 1:**
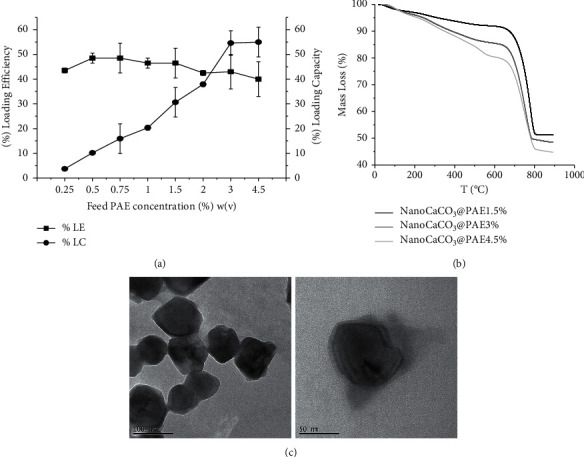
(a) Loading efficiency and loading capacity plot over PAE loading concentration by spectrophotometry analysis. (b) TGA traces of nanoCaCO_3_@PAE samples obtained from three different loading concentrations (1.5%, 3%, and 4.5% w/v). (c) TEM images at different magnifications of nanoCaCO_3_@PAE (deriving from PAE 3% w/v).

CaCO_3_ micro and nanoparticles are a versatile inorganic biomaterial for carriers' fabrication providing different applications in cosmetics, medicine, and agrifood industry [64, 65]. CaCO_3_ nanocrystals have already shown a good capacity to adsorb different organic species, both hydrophilic and hydrophobic. In particular, hydrophobic compounds, such as phospholipids and polyphenols, have been trapped with a loading efficiency between 80 and 100%. Furthermore, these delivery systems have widely improved bioactivity of adsorbed drugs, up to 100 times as described for the anticancer drug NVP-BEZ235 [59]. Their porous structure can be exploited also for surface functionalization to provide specific tags for microscopic analysis or cells targeting [58, 60]. These previous data have supported the utilization of nanoCaCO_3_ to enhance bioactivity of PAE that is rich of organic metabolites with a potential affinity with this nanoporous material. Loading efficiency has been evaluated quantifying free extract in supernatants as just described in Materials and Methods section. Standard curve has been obtained measuring UV adsorption at 260 nm. In Figure S1A, we can observe the adsorption spectra of PAE and nanoCaCO_3_@PAE suspension that showed the splitting of indicative peak, probably due to phytochemicals interaction with crystals surface. Standard curve by Origin software is shown in Figure S1B. First, loading efficiency was investigated, maintaining fix nanocrystals amount and varying PAE concentration. In our previous work, the phenol caffeic acid has shown an enhancement of LE from 60% to 80% increasing the loading concentration eight times. The plot in Figure 1(a) shows PAE LC and LE versus loading concentrations. The LE remains in the range 45–50% increasing the concentration sixteen times, confirming that this parameter not decisively affected PAE metabolites efficiency adsorption on nanoCaCO_3_. Second, washing supernatants were also quantified to calculate loading capacity. The LC peak has been reached at 3%w/v concentration. The LC trend indicated that the adsorbed substances at 3 and 4.5%w/v of PAE have been lost during washing, suggesting a weak physical-chemical adsorption and a saturation of binding sites. Organic layer of PAE is evident observing nanocrystals TEM images, which are shown in Figure 1(c). The nanoCaCO_3_@PAE lost the characteristic cubic shape of inorganic colloids, showing a more irregular morphology. TGA analysis of nanopowder after drying in the stove revealed loading efficiency tuning initial concentrations. First for all, both PAE and nanoformulation traces revealed phytochemicals degradation at temperatures above 50°C, i.e., the chosen temperature for samples drying in the stove. TGA plots are shown in Figure S2; we can observe a lower organic species adsorption starting from PAE 1.5% w/v than those from PAE 3% and 4.5% w/v. Samples deriving from 3% to 4.5% w/v PAE solutions showed a similar mass loss plot, suggesting the binding sites saturation of nanoCaCO_3_ surface. TGA plots of PAE and nanocrystals are shown in S2A, which visualize theplainly different thermal degradation of organic components and inorganic nanocarriers. Furthermore, nanoCaCO_3_@PAE@CH has a greater organic contribution than nanoCaCO_3_@PAE due to the polymeric coating, as can be deduced from the TGA traces reported in S1B.

This adsorption behaviour indicated that the main involved force is not the capillary one due to the nanoporous structure of particles. Therefore, chemical interactions drive extract adsorption, and they could also affect substances release. For this reason, release study has been supported by NMR analysis to obtain qualitative characterization.

**Figure 2:**
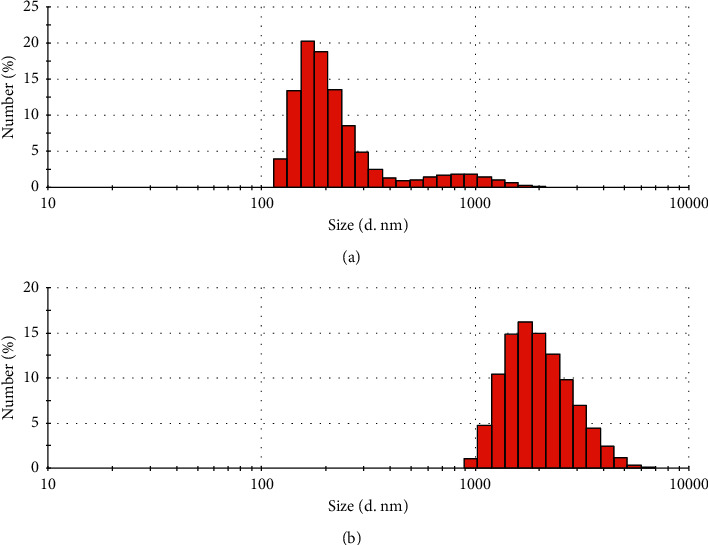
Size distribution plot by Number percentage of nanoCaCO_3_@PAE (a) and nanoCaCO_3_@PAE@CH (b) nanoformulations in water.

NanoCaCO_3_@PAE formulation resulted very stable, as indicated by negative *ζ*-potential (−23 ± 1 mV) and size distribution analysis that revealed an average hydrodynamic diameter of 490 nm with 0.4 PDI value. These DLS measurements suggested a reduction of nanocrystals aggregation in water, thanks to additional electrostatic repulsions from PAE chemicals. Size distribution plot by number percentage is shown in Figure 2(a), which is evident from the major particles' population in the range 100–500 nm. Chitosan coating has been investigated to tune PAE release and bioactivity of resulted nanoformulation. This biopolymer has been selected, thanks to its important features. It works as a good coating agent providing positive charge, and so, it is widely exploited for drug delivery systems fabrication [66]. Furthermore, chitosan has showed a great antimicrobial activity toward different pathogens [67]. We have recently demonstrated the strategic role of chitosan in the formation of stable and functional water-dispersible fosetyl-Al nanosuspension [68]. The increment of *ζ*-potential from −23 ± 1 mV to −9 ± 0.5 mV indicated the polymer coating on nanoCaCO_3_@PAE. However, this low absolute value of *ζ*-potential induced nanocrystals aggregation in water as shown by size distribution (Figure 2(b)). The PAE presence prevents further absorption of polymer, and for this, the surface charge inversion is not observed.

Following studies, about *in vitro* release and bioactivity of PAE nanoformulation, have been carried out comparing nanoCaCO_3_@PAE@CH and nanoCaCO_3_@PAE samples, respectively, with and without chitosan.

**Figure 3:**
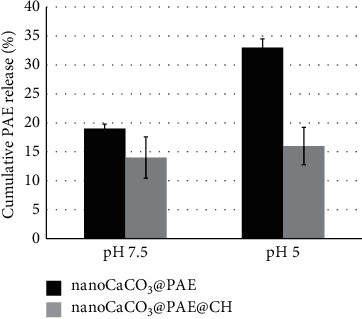
Cumulative PAE release from nanoformulations after 48 h incubation at pH 7.5 and 5.

In vitro release has been studied at pH 7.5 and 5, monitoring nanoCaCO_3_@PAE@CH and nanoCaCO_3_@PAE suspensions at 1 mg/ml from 3 h to 72 h, as described in Materials and Methods section. Both samples reached the plateau after 48 h under these experimental conditions. Nanoformulations composition, such as core/shell concentrations and surfactants utilization, principally affects extract LE and release [54]. In our case, the substantial differences have concerned pH exposition and presence of chitosan coating, as we can observe in the plot of cumulative release in Figure 3.

NanoCaCO_3_@PAE showed an increasing extract release after 48 h reducing pH, thanks to known susceptibility under acidic conditions of nanoCaCO_3_. Extract release at pH 7.5 is due to CaCO_3_ pKa of 9 that provides a slow dissolution allowing compounds release. PAE release at pH 5 for nanoCaCO_3_@PAE is double that for nanoCaCO_3_@PAE@CH. Chitosan coating protected adsorbed molecules and release did not change varying pH. This behaviour depends on chitosan solubility at extremely acidic conditions, so investigated pH values did not allow polymer dissolution and loaded substances delivery.

### 3.2. NMR Characterization and Release Monitoring

**Figure 4:**
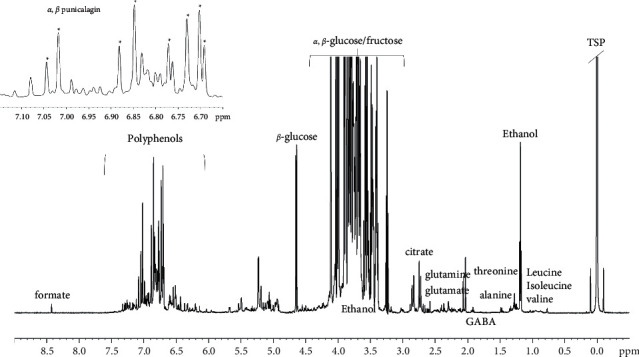
^1^H zgcppr NMR spectrum of PAE in D_2_O.

A widely used approach for the evaluation of fruit juice authenticity is the metabolic profiling [69–72]. Different analytical techniques have been used for this purpose including gas chromatography [71], liquid chromatography-mass spectrometry [72], Fourier transform infrared spectroscopy, and nuclear magnetic resonance (NMR) spectroscopy [70]. In particular, NMR spectroscopy has shown great potential as a detection technique in complex mixture analysis, mainly for the ability to detect multiple (10–100 s) metabolites at once without separation. This technique is nondestructive and can be quantitative (different from MS) but capable at the same time to allow metabolic profiles or “fingerprint” collection of the examined biological samples [73]. In this context, ^1^H NMR spectroscopy has been performed for a qualitative analysis of the PAE metabolic profile. The ^1^H zgcppr NMR spectrum of PAE, shown in Figure 4, is mainly characterized by amino acid (alanine, threonine, glutamine, and glutamic acid), organic acids (mainly citrate), sugars (identifiable *α* and *β* glucose signals, *α* and *β* form of fructose), and polyphenolic compounds (ellagitannins), as given in supplementary Table 1.

As reported, polyphenols, the predominant class of phytochemicals of pomegranate fruits, are responsible for the high benefits of pomegranate. The most abundant tannin found in pomegranate juice is punicalagin [74, 75]. Punicalagin, a member of the ellagitannin family, consists of a central glucose moiety existing in *a*- and *β*-anomeric forms esterified by ellagic acid, a gallic acid dimer, and gallagic acid, a dimer formed from ellagic acid [76]. Despite the spectral complexity (overlapping signals and multiple distortions) of PAE, the eight aromatic singlet resonances, corresponding to the *α* and *β*-anomeric forms (four for each anomer), of punicalagin were clearly identified (see S 3).

#### 3.2.1. Study of pH Dependence in the Release of PAE from NanoCaCO_3_

Since pH dependence is one of the main relevant physical variables in drug release applications, a ^1^H NMR release study of nanoCaCO_3_@PAE formulation, in water suspension, was performed. The release was followed for two weeks, at basic (pH 8.9) and acidic (pH 5.01) conditions, directly from nanosuspension resuspended in water (D_2_O). For comparable results, the same nanoCaCO_3_@PAE concentration was used for both the experiments. Only the chemical species released from nanoCaCO_3_ (metabolites) are detectable, and the solid phase does not contribute to the NMR spectrum.

**Figure 5:**
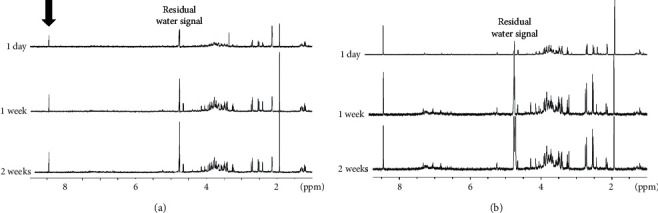
^1^H zgcppr NMR spectra of nanoCaCO_3_@PAE suspension at basic pH (a) and acid pH (b). The PAE release was followed for two weeks referred to TSP used as internal standard.

A gradual and increasing release of citrate, glucose, fructose, leucine, isoleucine, valine, and threonine was observed at basic pH, over two weeks of monitoring (Figure 5(a)). Similar release of polyphenolic component (6–8.5 ppm region in NMR spectrum) was not observed over the monitoring period. On the other hand, an enhanced release of all PAE metabolites, including the polyphenolic component, was clearly detected at acidic pH (Figure 5(b)). Therefore, NMR analysis indicated that nanoCaCO_3_ holds polyphenols more firmly than other components (sugars, amino-acids, and organic acids). This finding suggests the possibility to control the selective and gradual release of the different components of PAE using nanoCaCO_3_.

#### 3.2.2. Comparative Release Studies between NanoCaCO_3_@PAE and NanoCaCO_3_@PAE@CH

**Figure 6:**
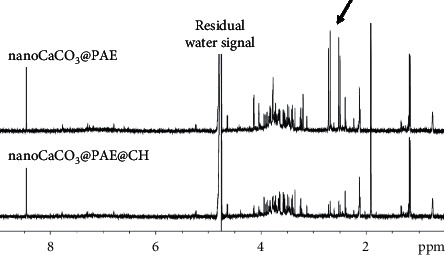
^1^H zgcppr NMR spectra of released PAE from nanoCaCO_3_@PAE and nanoCaCO_3_@PAE@CH at acidic pH (citrate release is indicated by black arrow).

The release of PAE from chitosan-coated nanoCaCO_3_ in respect to uncoated ones was also studied. The ^1^H NMR spectra of supernatants have been obtained performing the above-described *in vitro* release tests (acidic and basic conditions). A higher general release of PAE components, for both nanoformulations, was confirmed in the acidic with respect to the basic condition (data not shown). A general lower release for the chitosan-coated formulation (nanoCaCO_3_@PAE@CH) with respect to the uncoated (nanoCaCO_3_@PAE), in acidic condition, was observed (Figure 6). Moreover, NMR analysis revealed a specifically higher release of citrate from nanoCaCO_3_@PAE formulation with respect to nanoCaCO_3_@PAE@CH.

Both coated and uncoated nanoformulations showed a very slow release of polyphenols, slightly improved in acidic condition. Polyphenols may be considered as weak organic acids with the undissociated form prevailing at low pH and therefore promoting their cells membranes crossing. Rongai et al. demonstrated as antifungal activity of PAE could be due to the interaction between punicalagins and the pathogen membrane structure [28]. The investigated nanoformulations could further improve the PAE bioactivity through controlled and targeted release of specific metabolites such as punicalagin. Selective release of the different species composing pomegranate extract results, thanks to CaCO_3_ nanocrystals application. Specific bioactivity tests concerning antimicrobial, antiproliferative, and antioxidant properties have been also performed for this bioinorganic delivery system.

### 3.3. Antifungal Activity

Bioactive compounds from plants or agroindustrial residues have great potential as novel pesticides to control crops diseases, as discussed in the introduction section. We have tested PAE nanoformulations against two pathogenic fungi, *B. cinerea* and *F. oxysporum*. In previous works, PAE has shown a good antifungal activity for both these pathogens. It proposed an effect of PAE on fungi cell membrane, linked to biochemical properties of punicalagins [17, 28]. *B. cinerea* is one of the most important postharvest fungal pathogens causing significant losses in fresh fruits, vegetables, and ornamentals and showing significative fungicides resistance [77, 78]. PAE has a great inhibition capacity for *B. cinerea* mycelial growth, already after three days of treatment with the concentration of 0.75%w/v, and its effectiveness was also demonstrated for extension of strawberries shelf-life [28]. We investigated nanoCaCO_3_@PAE@CH formulation at different concentrations (0.25-0.5-1 %w/v) and nanoCaCO_3_@PAE at 1 %w/v. PAE and commercial fungicide at 1 %w/v have been used as controls. Four days after inoculum of *B. cinerea*, the not treated plates were entirely invaded by the fungus, while in plates with nanoCaCO_3_@PAE@CH, the mycelia growth peaked values between 40.1 at 0.25% and 17 mm at 1%. An intermediate antifungal activity (39.5 mm) was observed in plates with PAE at 1% concentration (Table 1). We have to consider that nanoformulation is composed of about half by weight of extract and the other half of nanoparticles, as indicated by adsorption data. Therefore, the nanoCaCO_3_@PAE@CH formulation at 1%w/v worked better than free PAE at comparable metabolites concentration. This result is due to the synergistic effect of nanocrystals and chitosan coating that improved phytochemicals availability and harmful interaction with fungal cells. In fact, a suspension of nanoCaCO_3_ at the highest tested concentration of 1%w/v induced low cells proliferation inhibition (20%). The antimicrobial effect was not observed for *F. oxysporum*, suggesting different involved biointeractions. *F. oxysporum* is soilborne pathogen with resistance to different synthetic fungicides that requires research of alternative and natural remedies. PAE at 0.5%w/v resulted inhibit 40% of *F. oxysporum* micelia growth after 5 days of incubation at 0.5%w/v [17]. As regards in vitro tests of this work, in the treated plates, the growth of the mycelium showed values between 40.5 (nanoCaCO_3_@PAE@CH at 0.25% concentration) and 18.2 mm (PAE 1%); the latter value did not show significant differences compared to the value of standard fungicide. Furthermore, there is no dose effect; in fact, by increasing the concentration of nanoCaCO_3_@PAE@CH, the growth of the mycelium did not decrease. At the concentration of 0.5%, the fungal growth was 40.4 mm, a value not significantly different from 40.3 observed in the plates treated with nanoCaCO_3_@PAE@CH at the concentration of 1% (Table 1). However, the highest concentration of nanoformulation referred to a free PAE concentration of 0.5 %w/v and carried out an inhibition of 20%. This data suggested that a major quantity of nanoCaCO_3_@PAE@CH is necessary to act against *F. oxysporum* cells, respect to *B. cinerea*. Moreover, Ren et al. have recently shown a good fungicide activity of chitosan, so this biopolymer could be further investigated to produce new nanotechnological tools against this phytopathogen [79].

**Figure 7:**
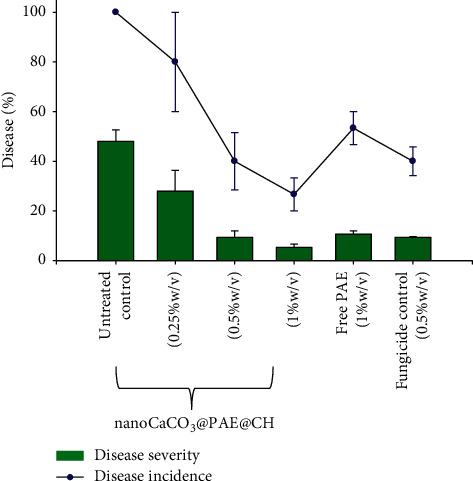
Increasing doses of nanoCaCO_3_@PAE@CH on disease incidence (DI) and disease severity (DS) of gray mould (*Botrytis cinerea*) on strawberry.

Chitosan-coated Au nanoparticles exhibited antifungal activity against two different strains of *F. oxysporum*; however, the effect dose/response remarkably varied depending on the strain [80]. This suggests that dosages, number of applications, and investigated strains play a crucial role in nanomaterials interactions with fungal cells. Chitosan solution has also showed good antimicrobial and filmogenic properties protecting from *B. cinerea* infection through specific defence mechanisms [81, 82]. In fact, the value of mycelial growth in nanoCaCO_3_@PAE 36.0 mm was much higher than 17.0 recorded in nanoCaCO_3_@PAE@CH. The recent bibliography reported the biomass materials exploitation and the smart strategy of micro/nanoencapsulation to control *B. cinerea* [83]. CaCO_3_-based nanoformulation has a very attractive potential as nanofillers in active food packaging. Recently, *Punica granatum* L. seed juice by-product was efficiently added as reinforcing and antimicrobial agent to gelatin films as a promising ecofriendly active food packaging material [84]. So, the in vivo test has been performed to control gray mould of strawberry caused by *B. cinerea*. NanoCaCO_3_@PAE@CH exhibited a great effect already from the lowest dose (Figure 7). By increasing the concentration of nanoformulation, both disease severity (DS) and disease incidence (DI) decreased: at 0.25% was detected DS and DI at 28% and 80%, respectively; both values are considerably lower than 48% and 100% obtained in the untreated control. A high control of gray mould in strawberry fruits was detected at concentrations of 0.5% and 1% with values of DS of 9.33 and 5.33%, while DI values are 40 and 26.66%, respectively. No statistically significant difference was found in treatments between nanoCaCO_3_@PAE@CH (0.5%) and PAE (1%), both for the DS value and for the DI value; however, as explained, nanoformulation concentration referred to nanocrystals amount plus extract amount.

Thus, nanoCaCO_3_@PAE@CH is very effective in reducing disease severity and incidence of gray mould. No significative effect for nanoCaCO_3_ alone (at 1%w/v) on disease severity and a low effect on disease incidence (20% less than untreated fruits) have been observed. Conventional pesticides formulations require high doses which often affect human and plants health. Problems of phytotoxicity and pollution, with contamination of foods, are recently solved by nanotechnologies application [85–88]. However, controlled-release systems are not always simple, and they usually involved materials and methods potentially toxic. The research in this field is yet an open topic [83]. The exploitation of nanoCaCO_3_ is strategic having assured biocompatibility and interesting features as drug delivery systems. It was expected to reduce applications and dosages of natural antimicrobial increasing bioactives efficacy tuning their release and targeting. Chitosan coating could have a crucial role in adhesion to fruits surface enhancing protection activity of PAE nanoformulation. Our data suggest the efficient application of nanoCaCO_3_@PAE@CH as novel postharvest treatments of strawberries to prevent *B. cinerea* infection during storage, as investigated for coatings of chitosan, silk fibroin, or methylcellulose [89]. It was assessed that *B. cinerea* infection induced already at early phases, upregulation of phytotoxins and ROS production which lead to great plants and fruits deterioration [90]. So, antioxidant activity of PAE and respective nanoformulations has been valuated too.

### 3.4. Antiproliferative Activity on Human Cell Lines

**Figure 8:**
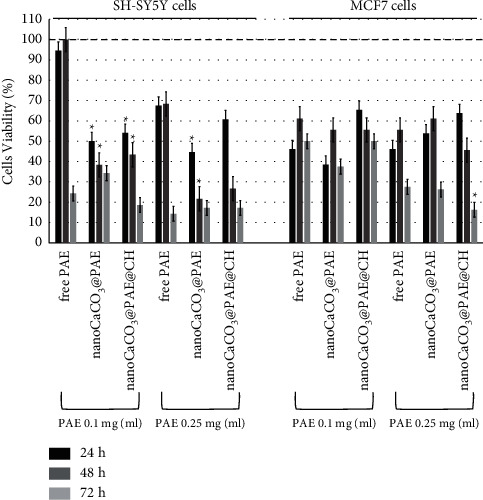
Cells viability by MTT tests for SH-SY5Y and MCF7 cells after 24-48-72 h of treatment with nanoCaCO_3_, free PAE, nanoCaCO_3_@PAE, and nanoCaCO_3_@PAE@CH at different concentrations. Percentage viability data referred to respective not treated control condition at each time point (100%, indicated by dashed line). Values represent mean from three independent experiments. Statistically significant value ^∗^*P* ≤ 0.05 is with respect to free PAE condition, from the *t*-test.

The antiproliferative effect has been studied through MTT assay on two human cancer cell lines, MCF7 and SH-SY5Y cells. Cells viability has been measured incubating cells for 24-48-72 h with free PAE, nanoCaCO_3_@PAE, and nanoCaCO_3_@PAE@CH suspensions at two concentrations, in order to study comparable metabolites concentrations.  Figure 8 shows reported cells viability percentage plot versus treatments over time.

Naked nanocrystals did not affect cells viability, as shown in previous works for MCF7 cell line [61]. Cytocompatibility has been also demonstrated for SH-SY5Y, as shown by plot in S4. Therefore, observed reduction of cells viability depend only on investigated extract. Free PAE at lower concentration reduced SH-SY5Y cells viability after 72 hours of treatment, instead of respective nanoformulations of about 50–60% already after 24 hours. A clear viability reduction has been observed enhancing PAE concentration at 0.25 mg/ml. Also, in this case, the treatment with nanoCaCO_3_@PAE and nanoCaCO_3_@PAE@CH carried out a higher cytotoxic effect after 24–48 hours; instead after 72 h, there were no evident differences in the cytotoxic effect between free extract and nanoparticles (at both concentrations). These data indicated the importance of nanocrystals for plasmatic membrane overcoming and uptake of phytochemicals, as shown for other hydrophobic compounds [58, 59]. MCF7 cells have been more affected by PAE and PAE nanoformulations than SH-SY5Y cells. Already after 24 hours, free extract reduced at 50% cells viability, without a significative difference between the two concentrations. Nanoformulation has a higher effect than respective free PAE treatment only after 72 hours and in presence of chitosan coating. Chitosan provided a less negative charge to nanoCaCO_3_@PAE that has allowed a more effective interaction with cell membrane. Our previous work has demonstrated that biofunctionalization of nanoCaCO_3_ has a crucial role in cellular internalization and uptake kinetic depends on cell lines [62]. These aspects could explain the difference in terms of incubation time and cell type. In particular, MCF7 cells showed increasing CaCO_3_ nanocrystals localization in lysosomes after 24 hours of incubation [61]. Lysosomes acid pH induced a stronger release of PAE substances than neutral/basic condition. This could explain the bioactivity of nanoCaCO_3_@PAE@CH after 72 hours of incubation. This experiment highlighted variability of the bioactive effect due to cell lines. Furthermore, an enhanced antiproliferative effect has been found for PAE adsorbed-nanoparticles, eventhough the gradual release of metabolites has been demonstrated by NMR and spectrophotometric analysis. The great effect of pomegranate extract on MCF7 cell line is consistent with previous research. PLGA–PEG nanoparticles increased extract cellular uptake and subsequently its anticancer effect [52]. Badawi et al. reported a very low IC_50_ for nanoencapsulated samples; however, their systems provided a synergic effect of pomegranate chemicals and stearic acid of solid lipid nanoparticles [53].

The concentration of 0.1 mg/ml has been selected for the following experiments of intracellular ROS production assay to evaluate the antioxidant activity of PAE, not inducing cell death.

### 3.5. Antioxidant Activity

Pomegranate is a rich source of many phenolic and hydrolyzable tannins compounds which provide a very high antioxidant activity. These phytochemicals contribute to the reduction of oxidative stress in diseased tissues providing antiproliferative, antiinvasive, and proapoptotic effects as shown in several in vitro and in vivo studies. Therefore, the antioxidant activity attributes to pomegranate extract a great potential as chemopreventive action is useful for nutraceutical production [91]. First, we studied in vitro antioxidant capacity of PAE and nanoformulations through DPPH scavenging assay. In Table 2, percentages of antioxidant capacity are reported. Extract samples refer to experiments with comparable quantity of PAE. Ascorbic acid, at the same concentration, has been used as positive control.

Chitosan coating did not affect DPPH scavenging. Nanoformulations lowering capacity depend on gradual release of phytochemicals as just demonstrated by NMR characterization; in particular, phenolics compounds have disclosed a strong interaction with CaCO_3_. Despite the slow release, adsorbed extract provided an in vitro antioxidant capacity of about 70%.

The scavenging capacity can be correlated to antimicrobial activity that is a bioactivity strongly dependent to target species [12]. This aspect has been observed for PAE nanoformulations against *B. cinerea* and *F. oxysporum* pathogens. PAE and derivates are promising bioactive products to control pathogenic contamination of fruits, vegetables, and other foods, thanks to the synergic effect of antioxidant and antimicrobial activities. We have previously demonstrated the efficient postharvest control of *B. cinerea* by nanoCaCO_3_@PAE@CH, which also showed a great antioxidant capacity. Furthermore, Mekawi et al. provided lyophilized pomegranate peel nanoparticles extract' antioxidant activity as an effective tool in foods stability and safety, reducing acrylamide formation in sunflower oils [92].

**Figure 9:**
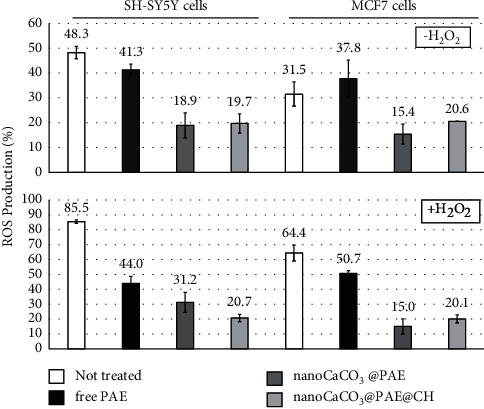
Percentage of ROS production of SH-SY5Y and MCF7 cells after 24 h of treatment with PAE, nanoCaCO_3_@PAE, and nanoCaCO_3_@PAE@CH at 0.1 mg/ml of extract and without and with previous H_2_O_2_ incubation.

We have investigated this bioactivity also in human cells. We have studied oxidative events in cells under two conditions, with and without external oxidative stress by H_2_O_2_. Cells have been treated for 24 hours with no cytotoxic extract concentration. Free extract has no antioxidant effect under normal condition; as we can observe in the top plot in Figure 9, differences have not been found between not treated and PAE-treated cells. Instead, both nanoformulations halved the production of intracellular ROS. No differences have been found depending on cell line. Oxidative stress induced by H_2_O_2_ is evident observing the percentage of ROS in control cells that is around 90%. In this stress condition, SH-SY5Y cells have been protected by PAE treatment, as indicated by the significative reduction of ROS production. NanoCaCO_3_@PAE and nanoCaCO_3_@PAE@CH were more efficient than the free formulation, as observed in the normal condition. The antioxidant capacity of nanoCaCO_3_@PAE is very high for MCF7 under oxidative stimulation.

Superoxide (O_2_^●−^) and nitric oxide (NO^●^) species have also been detected. Superoxide production in SH-SY5Y cells has been strongly reduced by treatment with nanoformulations, in both investigated conditions. No differences were observed between MCF7 cells treatments in normal condition, instead a clear effect of nanoformulations has been observed under external oxidative stimulation (data not shown). Nitric oxide has been produced in low quantities in both cell lines and also in stress condition, and free PAE did not affect NO^●^ production in all investigated conditions, instead nanoformulations have been almost set to zero NO in SH-SY5Y and MCF7 cells (data not shown).

These results demonstrated that PAE metabolites can inhibit intracellular oxidative processes. Adsorption on CaCO_3_ nanocrystals enhanced antioxidant activity in cell environment as shown for antimicrobial and antiproliferative activities. The resistance of cells to oxidative stress has been improved, thanks to nanocarriers ability to efficiently transport phytochemicals into cells. Encapsulation of phenolic extract in nanoparticles has showed a great ROS generation inhibition effect, both in noncancer and cancer cells and a good protection from degradation [93, 94]. Cancer cell function strictly depends on the intracellular levels of ROS which are involved in cell growth/proliferation, differentiation, protein synthesis, glucose metabolism, and inflammation process [95, 96]. So, scavenging propriety of pomegranate extract and its nanoformulations could have an interesting chemopreventive action. Furthermore, neurodegenerative diseases have been also related to oxidative stress [97]. Our results about SH-SY5Y cells treatment suggested a potential neuroprotective action of PAE that has been improved through nanoCaCO_3_ exploitation.

## 4. Conclusions

This work has demonstrated as controlled and targeted release of natural antioxidant and antimicrobial substances from pomegranate peel extract enhances their activity. Calcium carbonate is one of the most abundant biomineral in nature, and its micro and nanoparticles have been exploited in several applications ranging from industrial and biomedical fields. Calcium carbonate nanocrystals have been chosen because of their no toxicity and efficient efficacy in drug delivery for different biological applications. NanoCaCO_3_ has a great affinity toward different chemical species [58, 60]. PAE adsorption is very efficient, and its release could be tuned by changing pH that is a crucial factor for drug delivery systems, such as in cancer therapy. Polymeric coating is often used to functionalize carriers, so chitosan has been investigated to provide specific functions to PAE-adsorbed nanocrystals, obtaining nanoCaCO_3_@PAE@CH. We have found a higher PAE cumulative release at pH 5 than pH 7.5 that is prevented by chitosan coating. This trend has been confirmed by qualitative monitoring through NMR analysis. Chitosan has been investigated also for its intrinsic antimicrobial activity that has been studied for two phytopathogenic fungi. Furthermore, CaCO_3_ nanocarriers have been also exploited as efficient inorganic systems to control phytopathogen *X. fastidiosa*, implementing their applications spectrum [60]. A great increment of antimicrobial activity for nanoCaCO_3_@PAE@CH has been quantified for *B. cinerea*, a very aggressive postharvest pathogen. Efficient control of this fungus has showed treating strawberries. Antimicrobial activity of natural substances as PAE metabolites is often supported by scavenging capacity. DPPH assay has showed a very high antioxidant capacity of free PAE that is equal to that of ascorbic acid. Nanoformulations have preserved a great scavenging propriety of extract metabolites. Antioxidant activity has been also evaluated in intracellular condition, analyzing human cells through cytofluorometric assay. Neuroblastoma and breast cancer cells have been treated comparing free and adsorbed extract. NanoCaCO_3_@PAE and nanoCaCO_3_@PAE@CH significantly increased PAE inhibitory capacity of ROS, both in normal and stress condition. In this case, chitosan coating did not affect bioactivity. Interesting results have been obtained in antiproliferative tests too. Nanoformulations improved the PAE antiproliferative effect, both on SH-SY5Y and MCF7 cells. A high SH-SY5Y cells viability reduction has been induced already after 24 hours treating with CaCO_3_-based nanoformulations.

Our bioinorganic systems are derived from a smart integrated action between inorganic nanocrystals and organic chemicals with great biological proprieties. The proposed strategy allowed a simple and effective enhancement of PAE bioactivity as demonstrated by antimicrobial, antiproliferative, and scavenging capacity tests. NanoCaCO_3_@PAE@CH is a promising bioinorganic material in active food-packaging and in smart systems for postharvest pathogens control as well as systems for food deterioration prevention. CaCO_3_ nanocarriers application improves PAE anticancer activity suggesting the importance of continuing the strategy of encapsulation and controlled release. Finally, potential PAE chemopreventive and neuroprotective applications could be achieved through adsorption on nanocrystals, thanks to the enhanced intracellular ROS inhibition in neuroblastoma cells.

## Figures and Tables

**Table 1:** In vitro mycelial growth of *B. cinerea* and *F. oxysporum* by increasing doses of nanoCaCO_3_@PAE@CH. Values with different letters for each group are statistically different (LSD test, *α* = 0.05). Standard deviations of the means are indicated.

Treatment	Dose	Mycelial growth of *B. cinerea*		Mycelial growth of *F. oxysporum*	
		4^th^ day		4^th^ day	
		mm		mm	
Control		50.0	±0.00^a^	50.0	±0.00^a^
NanoCaCO_3_@PAE@CH	(0.25%)	40.1	±1.80^b^	40.5	±0.95^b^
NanoCaCO_3_@PAE@CH	(0.5%)	36.0	±2.92^b^	40.4	±0.81^b^
NanoCaCO_3_@PAE@CH	(1%)	17.0	±0.83^c^	40.3	±0.50^b^
NanoCaCO_3_@PAE	(1%)	36.0	±2.94^b^	40.0	±0.81^b^
PAE	(1%)	39.5	±0.50^b^	18.2	±1.70^c^
Fungicide	(0.5%)	16.8	±0.92^c^	17.2	±1.91^c^
			*F* = 385.76		*F* = 556.88
			*P* < 0.001		*P* < 0.001

**Table 2:** Percentages of antioxidant capacity by spectrophotometric DPPH assay testing samples at 3 mg/ml of PAE; data have been obtained from average of three experiments.

Sample	% antioxidant capacity
Ascorbic acid	94
PAE	92
NanoCaCO_3_@PAE	67
NanoCaCO_3_@PAE@CH	64

## Data Availability

The plots, tables and spectra used to support the findings of this study are included within the article and within the supplementary information file.

## Supplementary Materials

Supplementary Materials Supplementary Figure 1. UV spectra of PAE and resultant nanoCaCO_3_@PAE formulation, where punicalagins adsorption peaks are evident and standard curve from UV-absorption measurements at 260 nm peak from Origin program. Supplementary Figure 2. TGA analysis of PAE, CaCO_3_ nanocrystals, and nanoCaCO_3_@PAE deriving from PAE 3% w/v with and without chitosan coating. Supplementary Table 1. ^1^H NMR chemical shifts assignments of the metabolites present in pomegranate extract. Supplementary Figure 3. Expansion of the aromatic region derived from the ^1^H-^13^C HETCOR NMR detection of pomegranate PAE and ^1^H-^13^C NMR chemical shifts assignments of aromatic singlet resonances. Supplementary Figure 4. MTT test data on SH-SY5Y cells treated with two concentrations of nanoCaCO_3_.
