# Supplementary material for: Enhanced Bioactivity of Pomegranate Peel Extract following Controlled Release from CaCO3 Nanocrystals
Source: Bioinorg Chem Appl. 2022 Feb 12;2022:6341298. doi: 10.1155/2022/6341298 (PMC8858070; doi:10.1155/2022/6341298)
Supplement: Supplementary Materials — Supplementary Figure 1. UV spectra of PAE and resultant nanoCaCO3@PAE formulation, where punicalagins adsorption peaks are evident and standard curve from UV-absorption measurements at 260 nm peak from Origin program. Supplementary Figure 2. TGA analysis of PAE, CaCO3 nanocrystals, and nanoCaCO3@PAE deriving from PAE 3% w/v with and without chitosan coating. Supplementary Table 1. 1H NMR chemical shifts assignments of the metabolites present in pomegranate extract. Supplementary Figure 3. Expansion of the aromatic region derived from the 1H-13C HETCOR NMR detection of pomegranate PAE and 1H-13C NMR chemical shifts assignments of aromatic singlet resonances. Supplementary Figure 4. MTT test data on SH-SY5Y cells treated with two concentrations of nanoCaCO3. [file 6341298.f1.docx]

# Bioinorganic Chemistry and Applications

**Enhanced bioactivity of pomegranate peel extract following controlled release from CaCO_3_ nanocrystals**

## Francesca Baldassarre ^1,2^ *, Viviana Vergaro ^2^, Federica De Castro ^1^, Francesca Biondo ^1,2^, Gian Paolo Suranna ^2,3^, Paride Papadia ^1^, Francesco P. Fanizzi ^1^, Domenico Rongai ^4^ and Giuseppe Ciccarella ^1,2^ *

## ^1^ Department of Biological and Environmental Sciences, UdR INSTM of Lecce University of Salento, Via Monteroni, 73100 Lecce, Italy

## ^2^ Institute of Nanotechnology, CNR NANOTEC, Consiglio Nazionale delle Ricerche, Via Monteroni, 73100 Lecce, Italy

## ^3^ Department of Civil, Environmental, Land, Building Engineering and Chemistry (DICATECh), Politecnico di Bari, Via Orabona 4, Bari, 70125 Italy

## ^4^ CREA-IT PE - Research Centre for Engineering and Agro-Food Processing, via Lombardia, Pescara, Italy

## * Correspondence: francesca.baldassarre@unisalento.it (F.B.); giuseppe.ciccarella@unisalento.it (G.C.); Tel.: +39 0832 299469 (F.B.); +39-0832-319810 (G.C.)

## Abstract

Pomegranate peel extract is rich of interesting bioactive chemicals, principally phenolic compounds, which have shown antimicrobial, anticancer and antioxidative properties. The aim of this work was to improve extract bioactivity through the adsorption on calcium carbonate nanocrystals. Nanocrystals revealed as efficient tools for extract adsorption reaching 50% of loading efficiency. Controlled release of the contained metabolites under acidic pH has been found, as it was confirmed by quantitative assay and qualitative study through NMR analysis. Specific functionality of inorganic nanocarriers could be also tuned by biopolymeric coating. The resulting coated nanoformulations showed a great antimicrobial activity against *B. cynerea* fungus preventing strawberries disease better than a commercial fungicide. Furthermore, nanoformulations demonstrated a good antiproliferative activity in neuroblastoma and breast cancer cells carrying out a higher cytotoxic effect respect to free extract, confirming a crucial role of nanocarriers. Finally, pomegranate peel extract showed a very high radical scavenging ability, equal to ascorbic acid. Antioxidant activity, measured also in intracellular environment, highlighted a protective action of extract-adsorbed nanocrystals twice than free extract, providing a possible application for new nutraceutical formulations.

## Supplementary material

**Summary**

Supplementary figure 1 UV spectra of PAE and resultant nanoCaCO_3_@PAE formulation, where punicalagins adsorption peaks are evident and standard curve from UV-absorption measurements at 260nm peak from Origin program.

Supplementary figure 2 TGA analysis of PAE, CaCO_3_ nanocrystals and nanoCaCO_3_@PAE deriving from PAE 3 % w/v with and without chitosan coating.

Supplementary Table 1 ^1^H NMR chemical shifts assignments of the metabolites present in pomegranate extract.

Supplementary Figure 3 Expansion of the aromatic region derived from the ^1^H - ^13^C HETCOR NMR detection of pomegranate PAE and ^1^H - ^13^C NMR chemical shifts assignments of aromatic singlet resonances.

Supplementary figure 4 MTT test data on SH-SY5Y cells treated with two concentrations of nanoCaCO_3_.

Supplementary figure 1

**
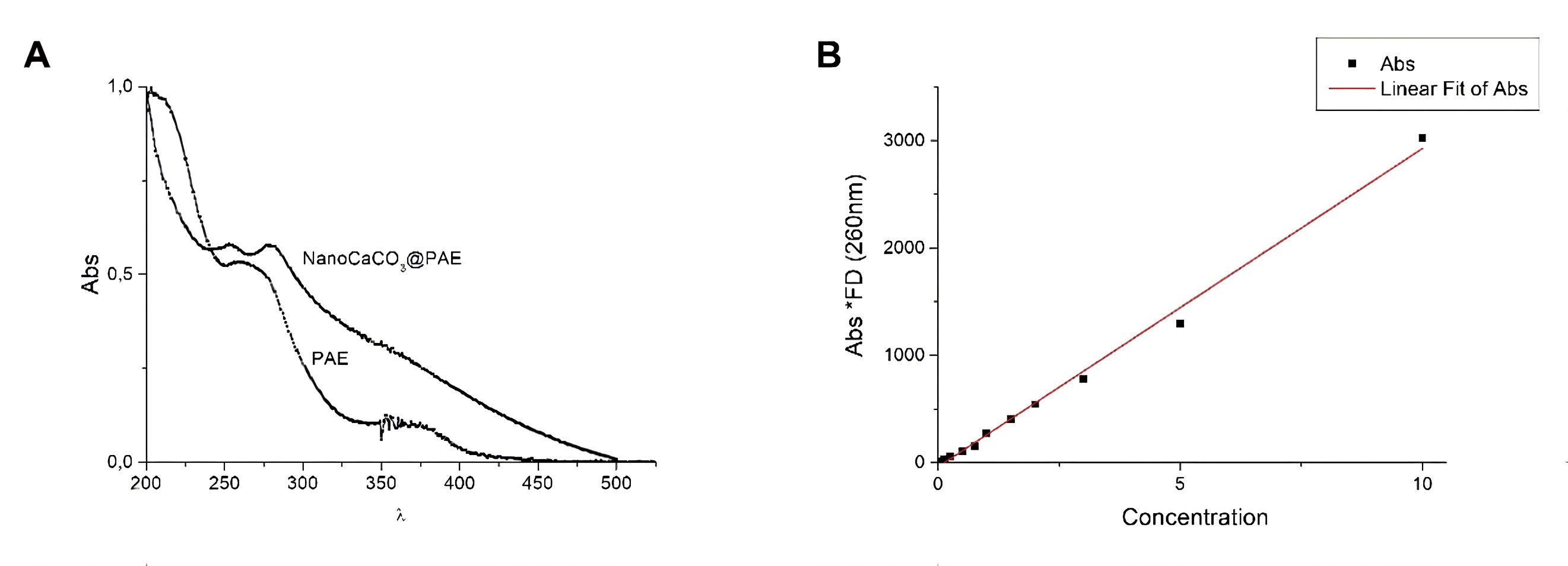
**

Supplementary figure 1: UV spectra of PAE and resultant nanoCaCO_3_@PAE formulation, where punicalagins adsorption peaks are evident (A). Standard curve from UV-absorption measurements at 260nm peak from Origin program (B).

Supplementary figure 2


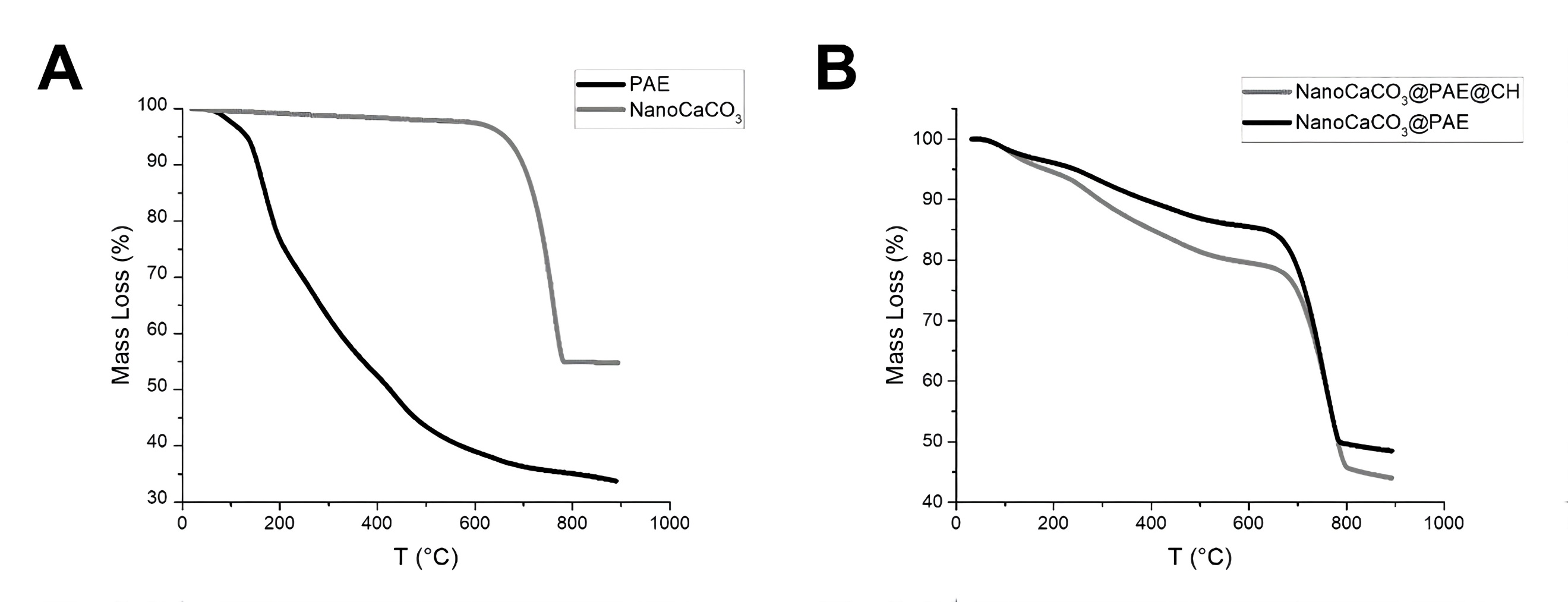


Supplementary figure 2: (A) TGA plot of PAE and CaCO_3_ nanocrystals; (B) TGA plot of nanoCaCO_3_@PAE deriving from PAE 3 % w/v, with chitosan coating (nanoCaCO_3_@PAE@CH) and without chitosan (nanoCaCO_3_@PAE).

Supplementary Table 1

Supplementary Table 1^: 1^H NMR chemical shifts assignments of the metabolites present in pomegranate extract. Spectrum has been acquired in D_2_O (pH 4.46; T 300K). Multiplicity: s(singlet); d (doublet); t (triplet); q (quartet); dd (doublet of doublets); m (multiplet).

| **Metabolites** | **^1^H NMR chemical shift ( δ, ppm )** |
| --- | --- |
| Alanine | 1.48 (d); 3.80 (m) |
| Ethanol | 1.18 (t); 3.66 (q) |
| Formate | 8.46 (s) |
| Glutamine | 2.15 (m); 2.45 (m); 3.77 (m) |
| Glutamate | 2.05 (m); 2.12 (m); 2.35 (m) |
| Isoleucine | 0.94 (t); 1.01 (d); 1.98 (m) |
| Leucine | 0.96 (d); 0.97 (d); 1.72 (m); 1.73 (m) |
| α - Glucose | 5.23 (d) |
| β - Glucose | 4.64 (d); 3.25 (t) |
| Citrate | 2.50 (dd) |
| Fructose | 4.12 (d) |
| α - Punicalagin | 6.7 (s); 6.73 (s); 6.88 (s); 7.03 (s) |
| β - Punicalagin | 6.69 (s); 6.77 (s); 6.92 (s); 7.08 (s) |
| Threonine | 1.35 (d); 3.58 (d); 4.24 (m) |
| Valine | 0.99 (d); 1.04 (d); 2.28 (m); 3.16 (d) |

Supplementary figure 3


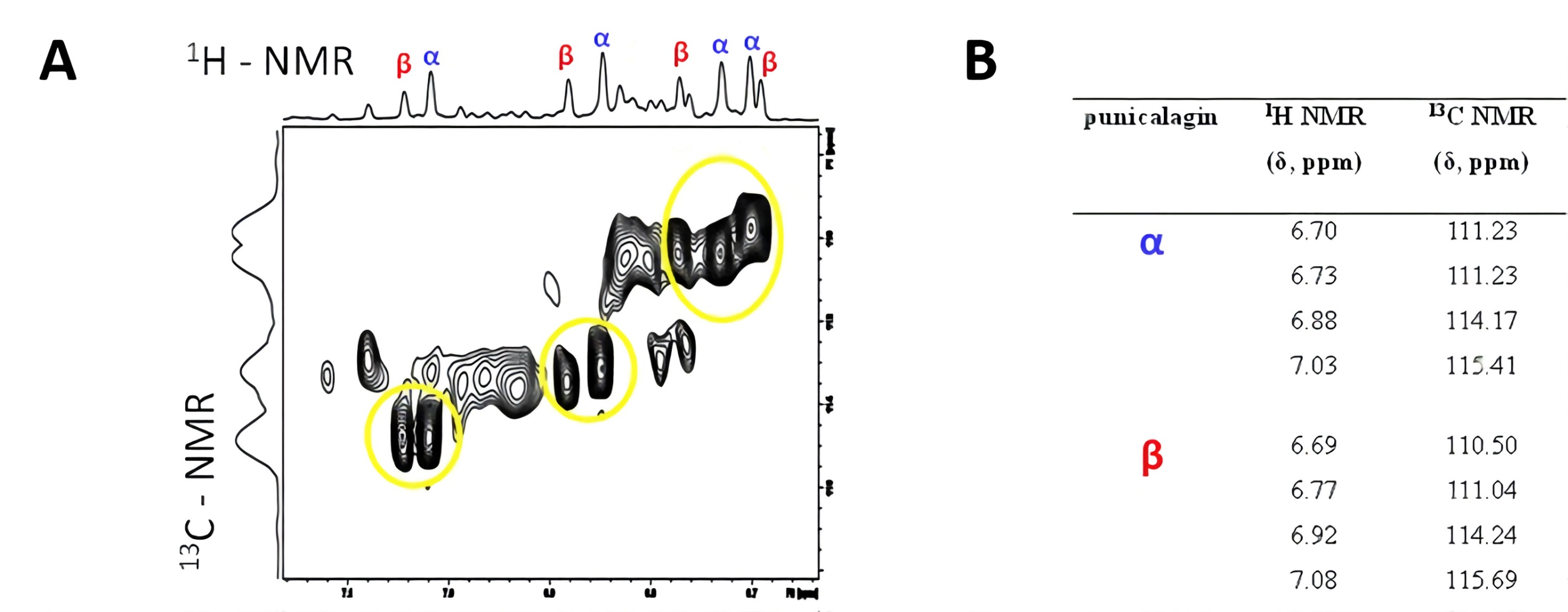


Supplementary figure 3: A) Expansion of the aromatic region derived from the ^1^H - ^13^C HETCOR NMR detection of pomegranate PAE. The aromatic protons of the two anomeric forms of punicalagin (α and β) are marked in yellow. B) ^1^H - ^13^C NMR chemical shifts assignments of aromatic singlet resonances of α- and β- punicalagin of pomegranate PAE.

Supplementary figure 4

**
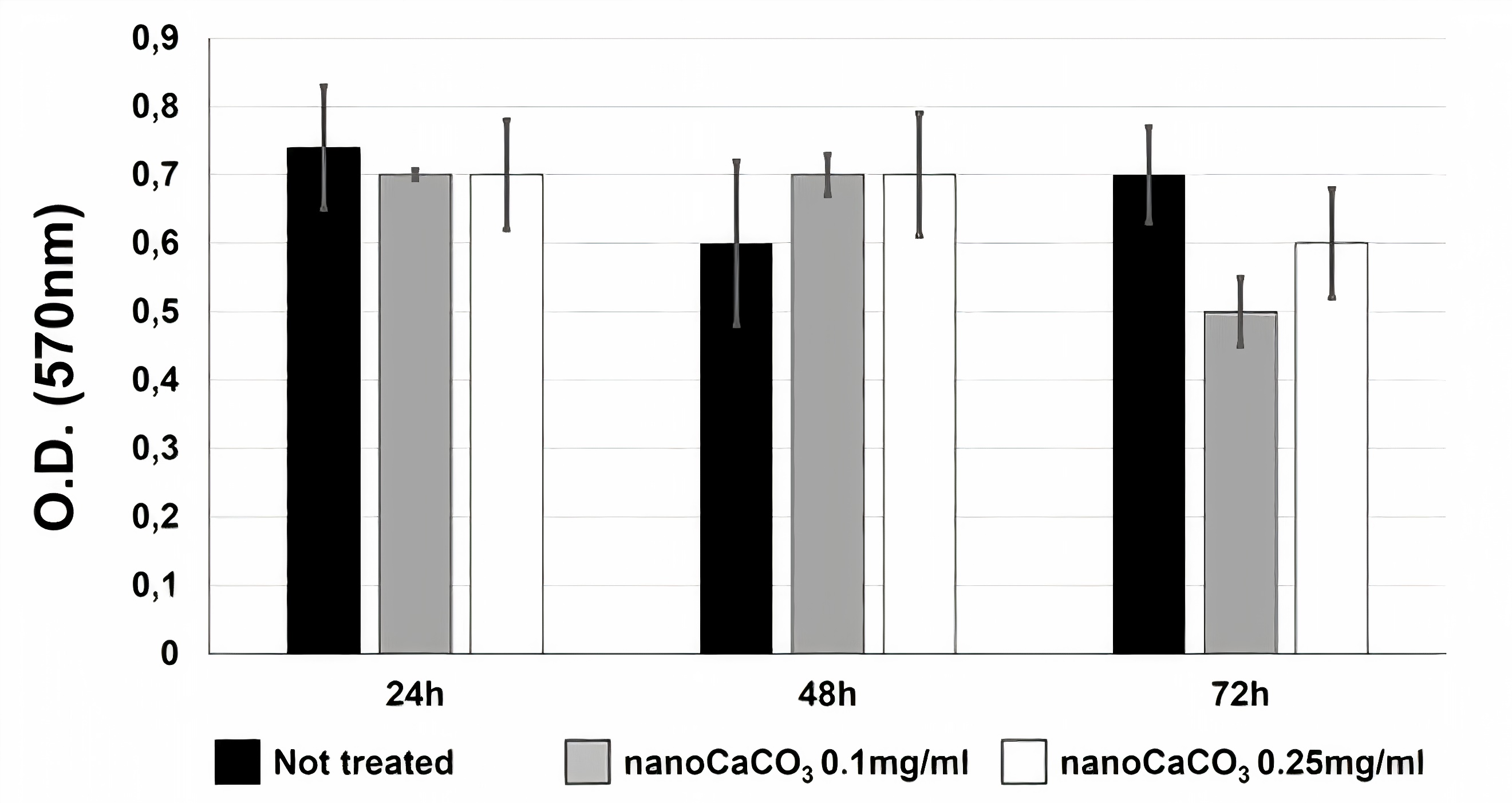
**

Supplementary figure 4: MTT test data on SH-SY5Y cells treated with two concentrations of nanoCaCO_3_.
